# The Scleroderma Patient-Centered Intervention Network Self-Management Program: Protocol for a Randomized Feasibility Trial

**DOI:** 10.2196/16799

**Published:** 2020-04-24

**Authors:** Marie-Eve Carrier, Linda Kwakkenbos, Warren R Nielson, Claire Fedoruk, Karen Nielsen, Katherine Milette, Janet Pope, Tracy Frech, Shadi Gholizadeh, Laura Hummers, Sindhu R Johnson, Pamela Piotrowski, Lisa Jewett, Jessica Gordon, Lorinda Chung, Dan Bilsker, Kimberly A Turner, Julie Cumin, Joep Welling, Catherine Fortune, Catarina Leite, Karen Gottesman, Maureen Sauve, Tatiana S Rodríguez-Reyna, Marie Hudson, Maggie Larche, Ward van Breda, Maria E Suarez-Almazor, Susan J Bartlett, Vanessa L Malcarne, Maureen D Mayes, Isabelle Boutron, Luc Mouthon, Fredrick Wigley, Brett D Thombs, SPIN Investigators

**Affiliations:** 1 Lady Davis Institute of the Jewish General Hospital Montreal, QC Canada; 2 Behavioural Science Institute, Clinical Psychology Radboud University Nijmegen Netherlands; 3 St Joseph’s Health Care London, ON Canada; 4 Scleroderma Society of Ontario Hamilton, ON Canada; 5 University of Western Ontario London, ON Canada; 6 University of Utah Salt Lake City, UT United States; 7 California School of Professional Psychology/Alliant Los Angeles, CA United States; 8 Johns Hopkins University School of Medicine Baltimore, MD United States; 9 Toronto Scleroderma Program Mount Sinai Hospital & Toronto Western Hospital Toronto, ON Canada; 10 Division of Neurosurgery Department of Surgery University of Toronto Toronto, ON Canada; 11 Private practice – Nutrition Hamilton, ON Canada; 12 Hospital for Special Surgery New York, NY United States; 13 Departments of Pediatrics, Biomedical Data Science, Psychiatry and Behavioral Sciences Stanford University Stanford, CA United States; 14 Simon Fraser University Burnaby, BC Canada; 15 University of British Columbia Vancouver, BC Canada; 16 NVLE Dutch patient organization for systemic autoimmune diseases Utrecht Netherlands; 17 University of Minho Braga Portugal; 18 Scleroderma Foundation Los Angeles, CA United States; 19 Scleroderma Canada Hamilton, ON Canada; 20 Instituto Nacional de Ciencias Médicas y Nutrición Salvador Zubirán Mexico City Mexico; 21 Department of Medicine McGill University Montreal, QC Canada; 22 McMaster University Hamilton, ON Canada; 23 Faculty of Behavioural and Movement Sciences Vrije University Amsterdam Netherlands; 24 University of Texas MD Anderson Cancer Center Houston, TX United States; 25 San Diego State University San Diego, CA United States; 26 University of Texas McGovern School of Medicine Houston, TX United States; 27 Université Paris Descartes Paris France; 28 Assistance Publique - Hôpitaux de Paris Paris France; 29 Department of Psychiatry McGill University Montreal, QC Canada; 30 Department of Epidemiology, Biostatistics, and Occupational Health McGill University Montreal, QC Canada; 31 Department of Psychology McGill University Montreal, QC Canada; 32 Department of Educational and Counselling Psychology McGill University Montreal, QC Canada; 33 See Acknowledgments

**Keywords:** feasibility studies, scleroderma, systemic, self-management, trial protocols

## Abstract

**Background:**

Systemic sclerosis (SSc), or scleroderma, is a rare disease that often results in significant disruptions to activities of daily living and can negatively affect physical and psychological well-being. Because there is no known cure, SSc treatment focuses on reducing symptoms and disability and improving health-related quality of life (HRQoL). Self-management programs are known to increase self-efficacy for disease management in many chronic diseases. The Scleroderma Patient-centered Intervention Network (SPIN) developed a Web-based self-management program (SPIN self-management; SPIN-SELF) to increase self-efficacy for disease management and to improve HRQoL for patients with SSc.

**Objective:**

The proposed study aims to assess the feasibility of conducting a full-scale randomized controlled trial (RCT) of the SPIN-SELF program by evaluating the trial implementation processes, required resources and management, scientific aspects, and participant acceptability and usage of the SPIN-SELF program.

**Methods:**

The SPIN-SELF feasibility trial will be conducted via the SPIN Cohort. The SPIN Cohort was developed as a framework for embedded pragmatic trials using the cohort multiple RCT design. In total, 40 English-speaking SPIN Cohort participants with low disease management self-efficacy (Self-Efficacy for Managing Chronic Disease Scale score ≤7), who have indicated interest in using a Web-based self-management program, will be randomized with a 3:2 ratio into the SPIN-SELF program or usual care for 3 months. Feasibility outcomes include trial implementation processes, required resources and management, scientific aspects, and patient acceptability and usage of the SPIN-SELF program.

**Results:**

Enrollment of the 40 participants occurred between July 5, 2019, and July 27, 2019. By November 25, 2019, data collection of trial outcomes was completed. Data analysis is underway, and results are expected to be published in 2020.

**Conclusions:**

The SPIN-SELF program is a self-help tool that may improve disease-management self-efficacy and improve HRQoL in patients with SSc. The SPIN-SELF feasibility trial will ensure that trial methodology is robust, feasible, and consistent with trial participant expectations. The results will guide adjustments that need to be implemented before undertaking a full-scale RCT of the SPIN-SELF program.

**International Registered Report Identifier (IRRID):**

DERR1-10.2196/16799

## Introduction

### Background

Rare diseases are chronic, disabling, and often life-threatening medical conditions. Individually, rare diseases affect fewer than 1 in 2000 people, but altogether, more than 1 in 15 people (6%-8%) have a rare disease [1,2]. The burden and impact on health-related quality of life (HRQoL) of most rare diseases are high, and in most cases, there is no therapy that cures or modifies the disease itself [3,4]. Psychological, educational, and rehabilitation interventions could contribute to improving function and alleviating distress for patients, and international rare disease plans have emphasized the need for effective disease management tools to complement basic medical care [5,6].

Self-management programs are widely disseminated and are known to increase patient self-efficacy for disease management and HRQoL in many common chronic diseases [7,8]. In rare diseases, patients must address unique challenges that are not part of generic self-management programs [9]. It is difficult, however, to develop, test, and disseminate self-management programs that meet the needs of patients with rare disease [10]. One reason is that the small number of patients makes it difficult to conduct robust clinical trials and effectively disseminate patient tools [10].

Systemic sclerosis (SSc, or scleroderma) is a rare autoimmune disease that affects the skin and internal organs, including the lungs, gastrointestinal tract, and cardiovascular system [11,12]. SSc is notable for the range of problems faced by people living with the disease, including limitations in physical mobility and hand function, pain, fatigue, sleep disturbance, depression, sexual dysfunction, and body image distress from disfiguring changes in appearance [10,13-16]. Self-management is complex in SSc and must address a wide range of concerns, such as skin care, gastrointestinal symptoms, and disfiguring changes in appearance, which are typically not part of generic self-management programs or self-management programs for more common rheumatic diseases [17,18].

One randomized controlled trial (RCT) comparing the effectiveness of an internet self-management program with an educational book was published recently, and two pre-post intervention studies of SSc self-management programs have been conducted [19-21]. Of the two pre-post studies, one described a mail-delivered program provided to 49 patients, and the other was a pilot study of an internet-based self-management program with 16 patients [19,20]. The small sample sizes and lack of control groups, however, did not allow conclusions to be drawn about effectiveness. Furthermore, the small number of patients involved in these studies underline the difficulty of conducting adequately powered research in a rare disease context. The published RCT compared the effects on self-efficacy of the same internet self-management program as described in the pilot study [20] with those of an educational book in a sample of patients with SSc from the United States (n=267). There were no statistically significant differences between the two groups, but patients were enthusiastic about the Wed-based program and its content [21]. Thus, robust evidence on the effectiveness and availability of accessible self-management tools for patients with SSc remains limited.

The Scleroderma Patient-centered Intervention Network (SPIN) [22], a collaboration of SSc research centers, clinicians, and patient organizations from Canada, the United States of America, Europe, Mexico, and Australia, was created to address this problem [10]. SPIN has assembled a large multinational patient cohort to collect longitudinal data on patient-reported outcomes in SSc and as a framework for embedding RCTs of electronic health interventions on an ongoing basis. To date, more than 2000 patients with SSc from 47 centers have been enrolled in SPIN’s Web-based cohort (currently approximately 1800 active participants).

SPIN’s Patient Advisory Board has prioritized the need for an SSc-specific disease management program. As such, the SPIN self-management (SPIN-SELF) program was designed by SPIN investigators based on successful self-management programs for more common diseases, informed by data on SSc-specific patient concerns and coping challenges, patient input obtained through a series of focus groups [9], and direct input from patients. The program includes multiple modules that focus on self-efficacy enhancing strategies and provide the knowledge, skills, and confidence essential to managing the physical, emotional, and social consequences of SSc. The SPIN-SELF program utilizes social modeling through educational videos of patients with SSc who describe their own challenges and what they have done to cope with living with SSc, as well as videos of patients and health professionals who teach key self-management techniques [23].

### Objective

We will conduct a full-scale RCT to assess the effectiveness of SPIN-SELF on improving self-efficacy and HRQoL. Before this, a feasibility trial of SPIN-SELF is being conducted to ensure the feasibility of the planned trial methodology and that the Web-based intervention is user-friendly and acceptable to trial participants [24-27]. The outcomes of this study will inform and guide adjustments to the Web-based SPIN-SELF program and the trial procedures that may need to be implemented before undertaking the full-scale RCT of SPIN-SELF.

## Methods

### Design and Setting

The SPIN-SELF feasibility trial is a parallel, two-arm, multicenter RCT that will be conducted via the SPIN Cohort (ClinicalTrials.gov number NCT03914781). The SPIN-SELF feasibility study is not meant for hypothesis testing or effect size estimation, as the sample size is not appropriate to do so, and testing hypotheses about effectiveness is discouraged in the context of feasibility trials [24-27].

#### The Scleroderma Patient-Centered Intervention Network Cohort Participants

The SPIN Cohort was developed as a framework for embedded pragmatic trials using the cohort multiple RCT (cmRCT) design. In the cmRCT design [28], participants enroll in an observational cohort with regular outcome measurement. Participants consent to (1) allow their data to be used for observational studies; (2) allow their data to be used to assess intervention trial eligibility and, if eligible, be randomized; (3) if eligible and randomized to the intervention arm of the trial, to be contacted and offered access to the intervention, and if eligible and randomized to usual care, to not notify them that they are involved in the trial usual care group and to use their regularly collected cohort data to evaluate trial outcomes. Trial eligibility can be assessed during regular SPIN Cohort assessments, which occur every 3 months, and trial outcomes can be obtained at the subsequent SPIN Cohort assessment 3 months later.

To be eligible for the SPIN Cohort, patients must be classified as having SSc based on 2013 American College of Rheumatology/European League Against Rheumatism criteria [29] confirmed by a SPIN physician; be ≥18 years old; be able to give informed consent; be fluent in English, French, or Spanish; and be able to respond to questionnaires via the internet. The SPIN Cohort is a convenience sample. Eligible SPIN Cohort patients are recruited at SPIN sites [22] during regular medical visits, and written informed consent is obtained. A medical data form is submitted on the Web by the site to enroll participants.

#### Scleroderma Patient-Centered Intervention Network-Self Feasibility Participants

For the SPIN-SELF feasibility trial, 40 English-speaking SPIN Cohort participants will be randomized with a 3:2 ratio to be offered to use the SPIN-SELF program or usual care for 3 months. Cohort participants will be eligible for the feasibility trial if they complete their SPIN Cohort measures in English, have low disease management self-efficacy (Self-Efficacy for Managing Chronic Disease Scale score [SEMCD]≤7 [30]), and have indicated high interest in using a Web-based self-management intervention (≥6 on 0-10 scale). Assessments of disease management self-efficacy and interest will occur as part of participants’ regular SPIN Cohort assessments.

### Procedure: Randomization, Allocation Concealment, Consent, and Blinding

**Figure 1:**
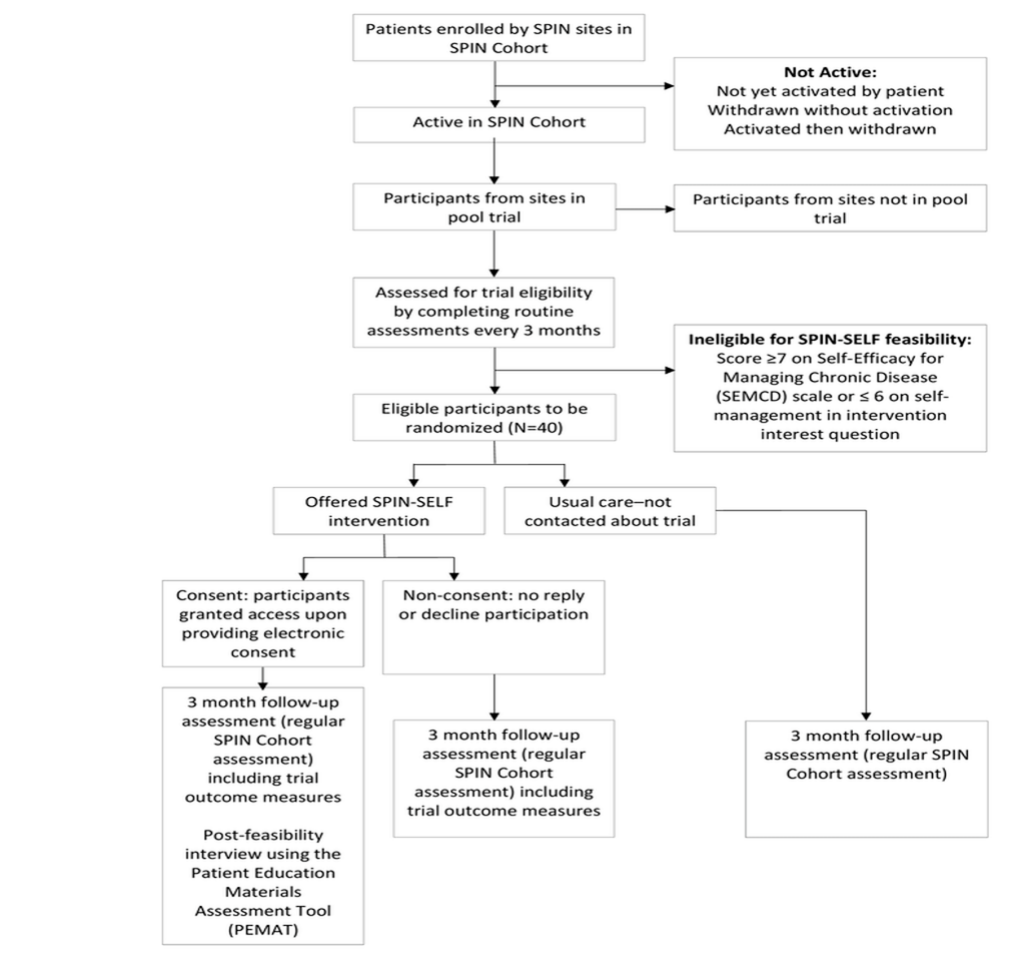
Scleroderma Patient-centered Intervention Network Cohort and feasibility trial flow diagram.

Randomization to be offered vs not offered to the SPIN-SELF intervention will occur at the time of cohort participants’ regular SPIN Cohort assessments. Eligible cohort participants, based on questionnaire responses, will be randomized automatically as they complete their regular SPIN Cohort assessments using a feature in the SPIN Cohort platform, which provides immediate centralized randomization and thus complete allocation sequence concealment. Participants randomized to be offered the intervention will receive an automated email invitation including a link to the SPIN-SELF program site and the SPIN-SELF feasibility study consent form. At initial log-in, they will be prompted to provide written consent to participate in the SPIN-SELF feasibility trial by verifying agreement with consent elements and providing their email address as the signature. Participants who consent will be automatically redirected to the introduction page of the SPIN-SELF program. Patients who log out before agreeing to the terms of the consent form will return to the consent page upon subsequent log-ins. Participants who accept the offer to use the SPIN-SELF program can use the weblink to enter the secure intervention site. SPIN personnel will also contact participants by phone, usually within 48 hours of sending the invitation email, to describe the study, review the consent form, and answer questions. Within the first 10 days, after the SPIN-SELF invitation email is sent, SPIN personnel will attempt to contact participants, up to a maximum of five times, to offer them more information about the study, to answer any questions they may have, and to help them consent or log in to the program. If a participant is still unreachable, a sixth and last attempt will be conducted by SPIN personnel to complete the call protocol at approximately 20 days postinvite. Email and phone technical support will be available to help participants with the consent process and to access and use the intervention site. See Figure 1 for SPIN Cohort participants' flow through the SPIN-SELF feasibility trial.

In pragmatic trials, participants are typically not blinded to intervention status, and possible biases are accepted as part of the response to being offered an intervention, as may occur in practice [31,32]. Disappointment bias, however, can occur in conventional trial designs when a participant enrolls in a trial to receive an intervention but is allocated to usual care [28,31]. For this reason, in the cmRCT design [28], participants who are not offered an intervention are not notified that they have not been offered the intervention. This replicates actual practice, where patients are not typically advised about treatments that are not options, and reduces the risk of disappointment bias [28,31,32]. All participants in the SPIN Cohort are aware that SPIN will conduct intervention trials and are routinely asked about potential interest in nine possible interventions as part of their regular cohort assessments but are not informed that any particular intervention may be available unless they are offered to try the intervention. Thus, participants who are offered the intervention are not blind to their status, whereas participants assigned to usual care are blind to their participation in the trial.

### Intervention and Comparator

The SPIN-SELF program was designed based on key tenets of behavior change that have been incorporated in successful self-management programs for chronic diseases [17,23,33,34], as well as input from focus groups of patients with SSc and SPIN’s Patient Advisory Board. Most self-management programs follow a similar format. They include multiple modules that focus on self-efficacy enhancing strategies and provide the knowledge, skills, and confidence essential for managing the physical, emotional, and social consequences of a disease. Patients are not given direct solutions to problems, but rather are taught problem-solving and management skills. Each module includes an educational component, teaching of skills, and a goal-setting component [17,33,34]. The SPIN-SELF program utilizes social modeling through educational videos of patients with SSc who describe their own challenges and what they have done to cope with living with SSc, as well as videos of patients and health professionals who teach key self-management techniques [23].

**Figure 2:**
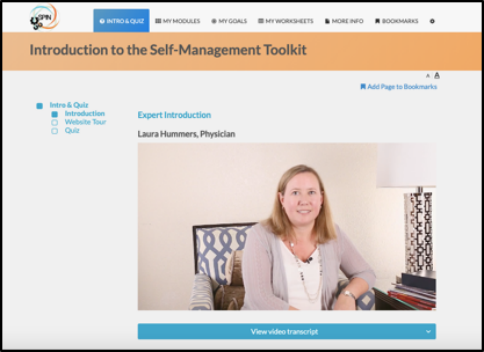
Scleroderma Patient-centered Intervention Network health care provider discussing SPIN-self-management Program.

**Figure 3:**
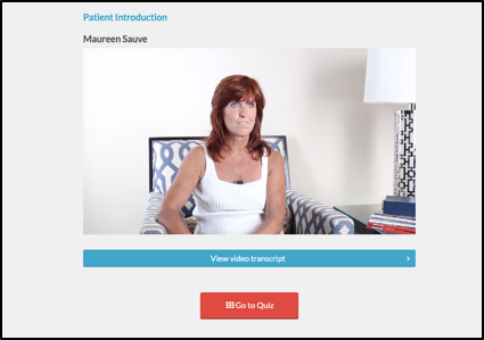
Patient discussing her experience with self-management.

**Figure 4:**
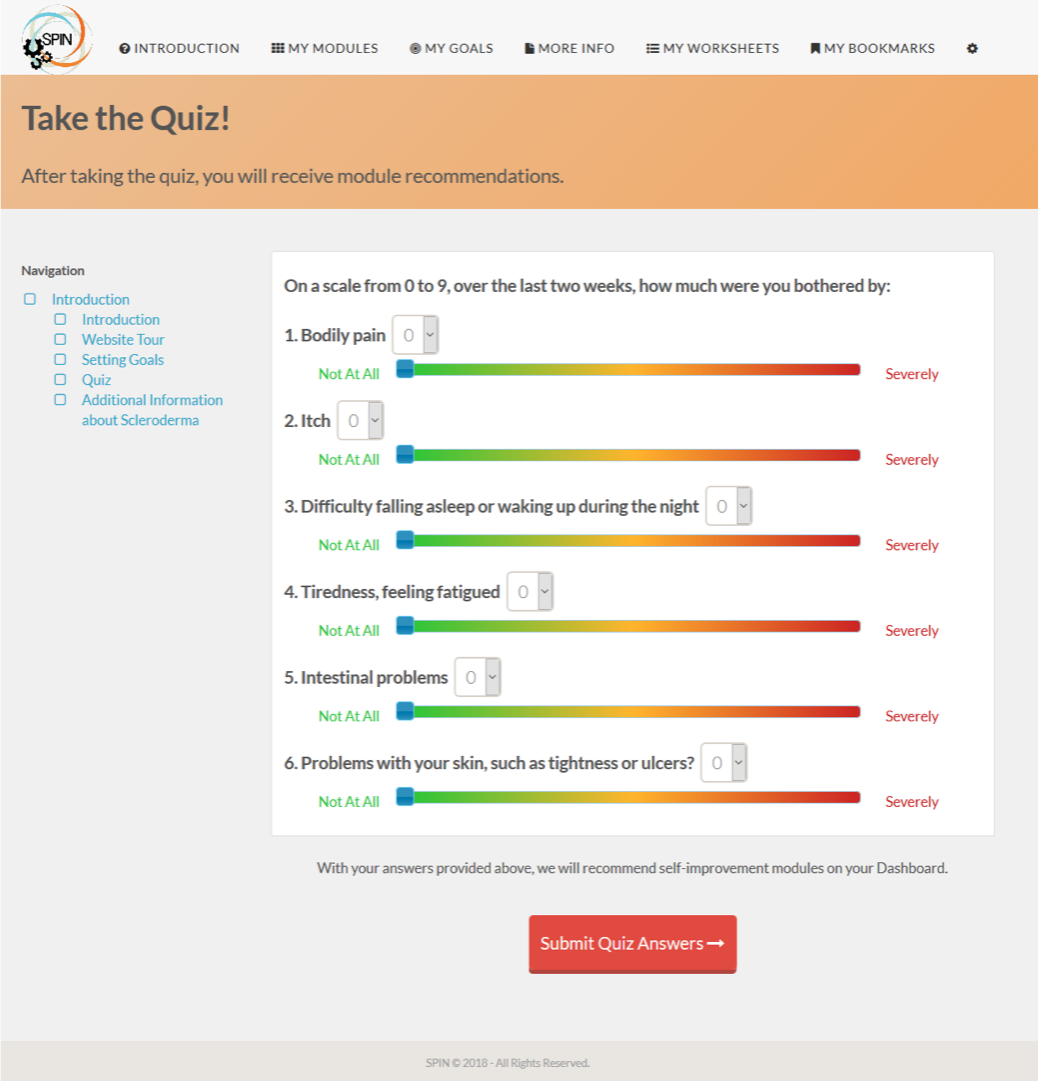
Nine-item quiz provides guidance to modules that are most relevant to a patients’ symptoms and disease management challenges.

After an introduction to self-management by a physician with expertise in the treatment of SSc (Figure 2), a patient shares her experience with learning how to become an efficient self-manager (Figure 3). Instructions on how to navigate the program are provided in a website tour video. Participants are then directed to a nine-item quiz that provides guidance to modules that are most relevant to a patient’s symptoms and disease management challenges (Figure 4).

**Figure 5:**
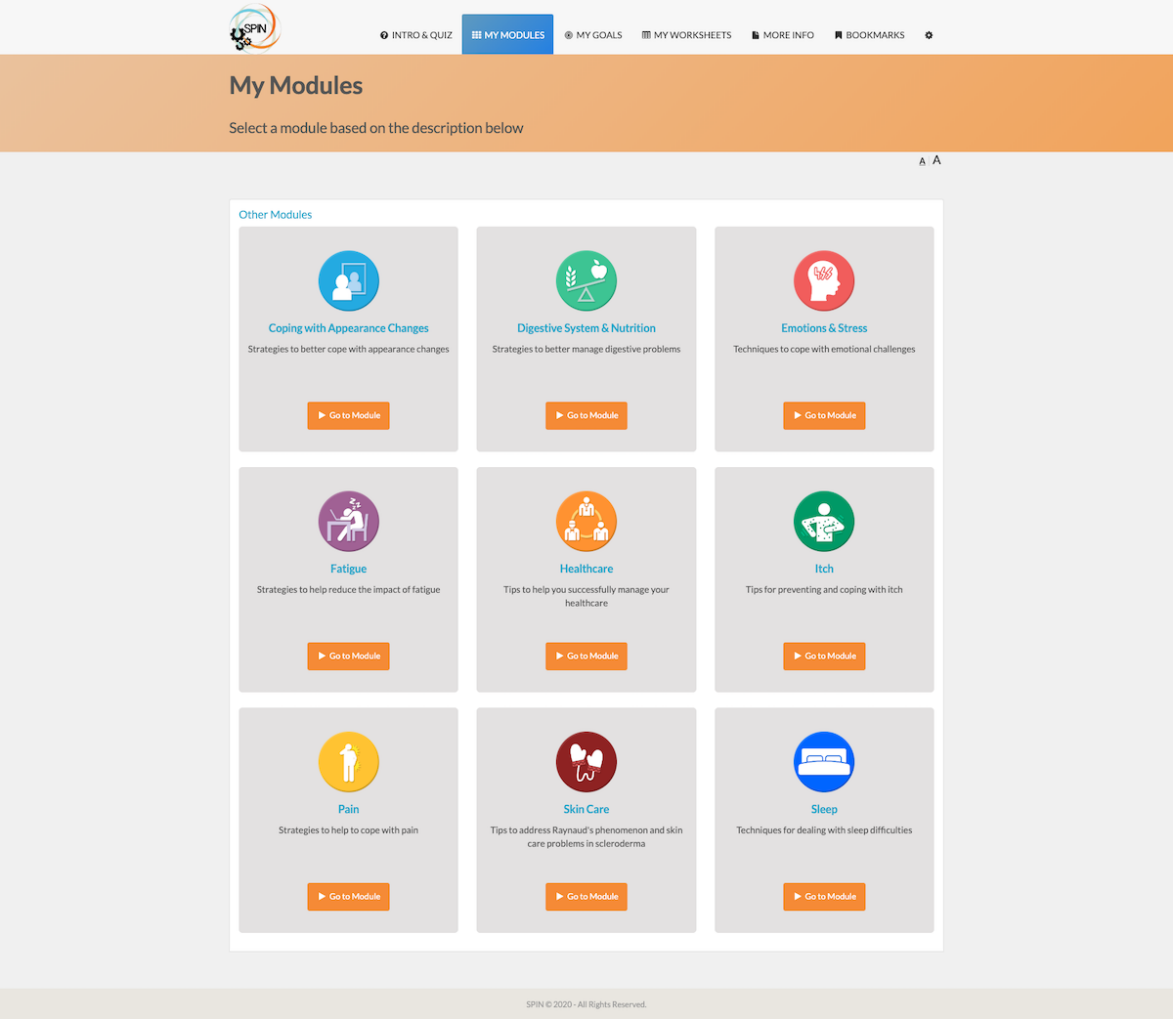
Menu of Scleroderma Patient-centered Intervention Network self-management modules.

The program’s nine modules focus on (1) coping with pain; (2) skincare, finger ulcers and Raynaud’s; (3) sleep problems; (4) fatigue; (5) gastrointestinal symptoms; (6) itch; (7) managing emotions and stress; (8) coping with body image concerns due to disfigurement; and (9) effective communication with health care providers. On the basis of the quiz score, the three modules that are most relevant for the patient will appear on top. Patients may access any modules, including the three identified as most relevant and all other modules; the other modules are shown underneath the three most relevant ones. Access to the nine modules in the program is unrestricted for the duration of the trial (Figure 5).

**Figure 6:**
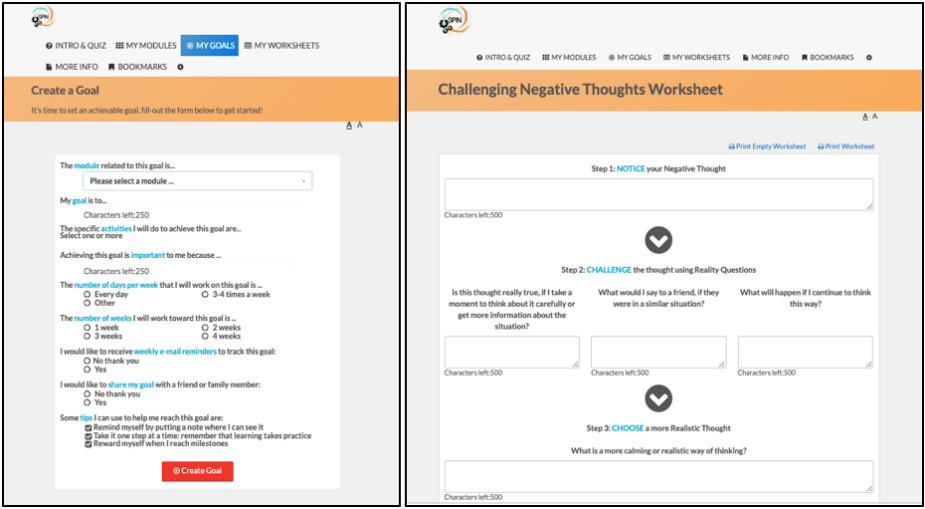
Tools to support key components of successful self-management programs, including goal-setting strategies, goal forms, and worksheets to learn how to integrate newly learned skills and techniques into a daily routine.

**Figure 7:**
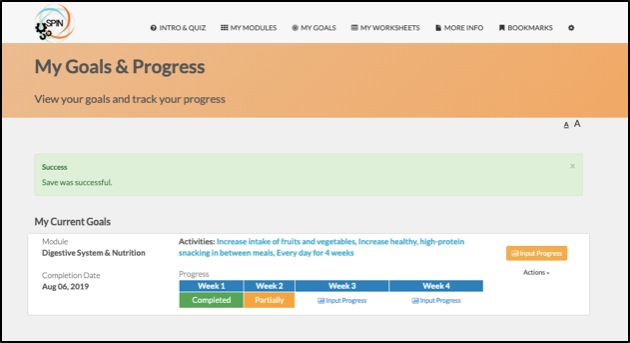
Goals and progress tracking tool.

**Figure 8:**
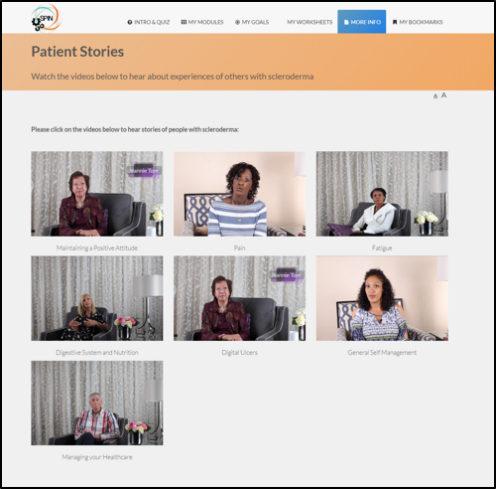
Patient stories page.

In addition to core modules, the SPIN-SELF program includes tools to support key components of successful self-management programs, including goal-setting strategies, goal forms, and worksheets to learn how to integrate newly learned skills and techniques into a daily routine [35] (Figure 6). For each goal that patients set, it is possible to input weekly progress, to share goals and progress with friends and family, and receive email reminders (Figure 7). Under the More Info section, general information on SSc can be found, in addition to patient stories of experiences with tips to improve self-management (Figure 8).

The program utilizes an engaging and easy-to-navigate Web interface. Favourite pages can be bookmarked for easy access, and text can be enlarged on every page. Scripts are available for each video.

### Outcomes and Measurement

The primary aim of the SPIN-SELF feasibility trial is to collect data related to the study’s *process* to assess the feasibility of the steps that need to take place as part of the full-scale RCT; *required resources and management* (eg, personnel and data management issues); and *scientific aspects* (outcome assessments). Data will be used to determine whether it is feasible to conduct the main study or whether changes need to be made before conducting a full-scale RCT of the SPIN-SELF program. The feasibility trial outcomes related to the process and resources will be assessed throughout the feasibility trial, and patient feedback will be obtained at 3 months postrandomization.

#### Process and Resources

Information to be collected includes (1) the proportion of SPIN Cohort participants who meet eligibility criteria, (2) proper functioning of automated eligibility and randomization procedures, (3) the proportion of eligible participants randomized to be offered the SPIN-SELF intervention who accept the offer and consent to participate, (4) completeness of Web-based data collection for each trial arm at 3-month follow-up, (5) completeness of the intervention usage log data, (6) ability to successfully link data coming from the SPIN Cohort and SPIN-SELF platforms, (7) rate of completion of trial outcome variables; (8) personnel requirements to call enrolled participants and help them to consent and use the SPIN-SELF program, (9) other challenges for study personnel, and (10) technological performance of the Web-based SPIN-SELF program.

#### Participant Use and Acceptability of Scleroderma Patient-Centered Intervention Network-Self Program

Usage of the SPIN-SELF program modules among participants in the intervention arm will be examined via intervention usage data. These data will provide detailed information on the number of log-ins, the number of modules accessed, goals set, as well as time spent on each webpage. In addition, at 3 months postrandomization, qualitative interviews will be conducted with participants in the intervention arm to assess user acceptability and satisfaction. The semistructured interview will be guided by items of the Patient Education Materials Assessment Tool for audiovisual materials [36] and will address topics related to usability, understandability, organization, and clarity. Participant feedback from these interviews will inform any changes necessary to improve the SPIN-SELF program before conducting a full-scale RCT. See Multimedia Appendix 1.

#### Full-Scale Trial Measures

The objective of the SPIN-SELF full-scale RCT is to evaluate the effect of being offered access to SPIN’s Web-based self-management program, in addition to usual care, on disease management self-efficacy and functional health outcomes for patients with SSc with low disease management self-efficacy, compared with usual care alone. In this feasibility study and the full-scale RCT, outcome measures that are routinely assessed as part of the SPIN Cohort assessments every 3 months will be evaluated. For the present feasibility study, we will assess variability in outcome measures and completion rates of outcome variables.

The primary outcome for the full-scale SPIN-SELF trial is *disease management self-efficacy,* which will be evaluated using the SEMCD [30]. The 6-item SEMCD scale measures confidence in the ability to manage fatigue, pain, emotional distress, and other symptoms, to do things to reduce illness impact other than taking medication and to carry out tasks and activities that may reduce the need to see a doctor. Items are rated on a 1 (*not confident at all*) to 10 (*totally confident*) scale. The total score is the mean of all items [30]. The SEMCD has been validated for measuring self-efficacy in patients with SSc through the SPIN Cohort [37]. Mean SEMCD scores of current SPIN Cohort patients who meet SPIN-SELF eligibility criteria is 5.1, which is similar to baseline scores in previous successful trials of self-management programs in other diseases [30].

*Patient-reported health status* will be measured using the 29-item Patient-Reported Outcomes Measurement Information System (PROMIS-29) profile version 2.0 scale. The PROMIS-29 measures eight domains of health status with 4 items for each of seven domains (physical function, anxiety, depression, fatigue, sleep disturbance, social roles and activities, pain interference) plus a single item for pain intensity. Items are scored on a 5-point scale (range 1-5), with different response options for different domains, and the single pain intensity item is measured on an 11-point rating scale. Higher scores represent more of the domain being measured, that is, better physical function and ability to participate in social roles and activities, but higher levels of anxiety, depression, fatigue, sleep disturbance, pain interference, and pain intensity. Total raw scores are obtained by summing item scores for each domain, which are converted into T-scores standardized from the general US population (mean 50, SD 10). The PROMIS-29 version 2.0 has been validated in SSc[38,39]

### Sample Size

Guidance on the appropriate sample size for feasibility trials varies substantially in the published literature, with rules-of-thumb varying from 12 to 30 or more per trial arm [40,41]. To ensure that we collect sufficient quantitative and qualitative outcome data to meet our feasibility objectives and guide the next study phase, we will include a total of 40 SPIN Cohort participants in this feasibility trial. We will randomize using a 3:2 ratio to get a sufficiently large number of patients in the intervention arm. In a previous feasibility trial of a SPIN hand exercises intervention, the rate of acceptance of the offer was approximately 60% [42].

### Data Collection, Storage and Sharing

Outcome measures are completed through the participants’ regular SPIN Cohort assessments. The SPIN Cohort uses a secure electronic data management platform designed and managed by the *Information Management Services of the Centre for Clinical Epidemiology, Jewish General Hospital, Montreal*. Separate from the SPIN Cohort portal, an encrypted database has been created for the SPIN-SELF program, which includes an identification number for intervention participants to link to SPIN Cohort data and their usage log information.

### Data Analysis

The primary data analysis will present a description of feasibility outcomes, including participants’ eligibility and recruitment and numbers and percentages of participants who respond to follow-up measures. Use of the internet intervention will be described by presenting the frequency of log-ins and the time spent on the SPIN-SELF program. Analysis of outcome measures will include the completeness of data and the presence of floor or ceiling effects. Descriptive statistics will be used to provide means and SDs for the measures. Qualitative information on participants’ experience using the SPIN-SELF intervention will be used to interpret acceptability related to the content, webpage visuals, and navigation and make any necessary changes to the intervention. Information related to the required resources and management of the program during feasibility will inform any necessary changes to intervention or trial procedures.

### Data Monitoring

The feasibility trial will be overseen by the SPIN Steering Committee, along with the trial investigators. The Steering Committee will provide scientific direction for the feasibility of the RCT. Routine monitoring of data quality will be handled by the SPIN director, in conjunction with trial investigators. The Steering Committee will be updated on progress during and after the trial.

### Adverse Events

The risk of adverse events occurring as a consequence of the SPIN-SELF program is very low. The only risks of participation in the SPIN-SELF feasibility study may be the possible unease or discomfort that some may experience resulting from viewing videos of others living with SSc, from answering questions about their SSc, or from reading the content about SSc in the program. Nonetheless, adverse events will be assessed via interviews and open-ended questions. Any events reported will be discussed with clinical members of the team, and referrals to local SPIN physicians will be made as necessary. Any serious adverse events that occur will also be reported to the ethics committee.

### Ethics and Trial Registration

Ethics approval for the SPIN-SELF feasibility trial has been obtained from the research ethics committee of the Jewish General Hospital, Montreal, Canada. The SPIN-SELF feasibility study was registered before participant enrollment (NCT03914781) and will be reported in accordance with standards articulated in the Consolidated Standards of Reporting Trials (CONSORT) extensions for randomized pilot and feasibility trials [27] and the draft CONSORT extension for trials using cohorts and routinely collected health data, which is forthcoming [43].

## Results

Enrollment of the target number of 40 participants occurred between July 5, 2019, and July 27, 2019. By November 25, 2019, data collection of trial outcomes was completed. Data analysis is underway, and results are expected to be published in 2020.

## Discussion

The SPIN-SELF program may improve self-efficacy for disease management and HRQoL in patients with SSc. This feasibility study will ensure that trial methodology is robust, feasible, and consistent with participant expectations [24-27]. Results will guide any changes that need to be implemented before conducting a full-scale RCT to test the effectiveness of the SPIN-SELF program. If effective, it will be made available through patient organizations around the world to support people in their efforts to cope with living with SSc.

## Appendix

Multimedia Appendix 1 Interview guide assessing usability, understandability, organization, and clarity of the Scleroderma Patient-centered Intervention Network self-management program.

Multimedia Appendix 2 Peer-reviewer report by Canadian Institutes of Health Research.

## Abbreviations

cmRCT: cohort multiple RCT
CONSORT: Consolidated Standards of Reporting Trials
HRQoL: health-related quality of life
PROMIS-29: Patient-Reported Outcomes Measurement Information System
RCT: randomized controlled trial
SEMCD: Self-Efficacy for Managing Chronic Disease Scale score
SPIN: Scleroderma Patient-centered Intervention Network
SPIN-SELF: SPIN self-management
SSc: systemic sclerosis
