# Supplementary material for: The Scleroderma Patient-Centered Intervention Network Self-Management Program: Protocol for a Randomized Feasibility Trial
Source: JMIR Res Protoc. 2020 Apr 24;9(4):e16799. doi: 10.2196/16799 (PMC7210498; doi:10.2196/16799)
Supplement: Multimedia Appendix 1 [file resprot_v9i4e16799_app1.docx]

MULTIMEDIA APPENDIX 1

**SPIN-SELF PROGRAM PATIENT INTERVIEWS**

PROCESS

1. Did the initial invitation email provide you with the information you needed to understand how to sign up for the study?

Yes. No.

If No: What information was missing?

2. Did you find the follow-up telephone call you received within 48 hours of the invitation email to be helpful?

Yes. No.

If No: Why not?

PURPOSE

3. Did you understand the objective of the SPIN-SELF program?

Yes. No.

If No: How could the objective be clarified?

4. Did you find the information provided in the SPIN-SELF program relevant to you?

Yes. No.

If No: How could the information provided be made more relevant to you or other scleroderma patients?

WORDS AND LANGUAGE

5. Did you find that the program used common, everyday language that was easy to understand?

Yes. No.

If No: Can you give an example of concept(s) or word(s) that you did not understand?

6. Did you understand all the medical terms or, if not, were they clearly explained in the SPIN-SELF program?

Yes. No.

If No: Can you give an example of medical term(s) that you did not understand?

CONTENT, ORGANIZATION, NAVIGATION

7. Did you find that the SPIN-SELF program was broken down into manageable chunks or sections?

Yes. No.

If No: Which parts of the content weren’t broken down into manageable chunks or sections and how could we improve them?

8. Did you find the different pages or sections of the program clearly labelled?

Yes. No.

If No: What section(s) could be more clearly labeled?

9. Did you find it easy to navigate through the intervention and to understand where to go next?

Yes. No.

If No: How could the different steps to navigate the intervention be more clearly explained?

10. Did you consult the “More info” tab (Scleroderma 101, Patient Stories)?

Yes. No.

If No: Why not?

11. Did you experience any technical difficulties while using the program?

Yes. No.

If Yes: What type of technical problems? Did you request assistance from the SPIN team? If you did, was the SPIN team able to help you resolve them?

12. Did you use the website tour?

Yes. No.

If Yes: Was it helpful to learn to navigate the website? Why or why not?

13. Did you use the “My Bookmarks” feature?

Yes. No.

If Yes: Did you find it helpful for easily navigating to the pages you wanted? Why or why not?

LEARNING AIDS

14. Did the fact that the program was introduced by scleroderma experts and patients make the program more credible and relatable?

Why or why not?

15. Did you understand how to correctly use the techniques explained in the modules?

Yes. No.

If No: What would have helped you better understand how to correctly perform the techniques?

16. Were you able to clearly understand the people speaking in the videos?

Yes. No.

If No: Why couldn’t you understand the words in the videos? (e.g. too fast, too soft, mumbling, accent)? Are there any videos in particular that were more difficult to understand than others? If yes, which one(s)?

17. Did you look at the video transcripts?

Yes. No.

If Yes: Were the video transcripts helpful to you? Why or why not?

ACTIONABILITY (Worksheets, Goal-setting, Motivation)

18. Did you use the worksheets?

Yes. No.

If No: Why not? What could have been a better tool?

19. Did you set goals for yourself using the “My Goals” feature?

Yes. No.

Why or why not?

20. Did you use the option to share your goals with friends and family via email?

Yes. No.

If Yes: Did the option to share your goals with friends and family via email help you stick with your goals? Yes. No.

If No: What other motivational feature(s) might have been more helpful?

21. Did you incorporate the tools and techniques you learned into your planned routine and stick with it?

Yes. No.

If No: What were some obstacles you faced when trying to incorporate the tools and techniques into your routine? How could the SPIN-SELF program have helped you to overcome these obstacles?

22. Did you use the feature to track your progress?

Yes. No.

If Yes: Did having the option to track your progress week after week encourage you to continue performing the techniques? Yes. No.

If No: Why not? Did you use any other way to track your progress? If so, what did you do?

23. Did you set email reminders for yourself?

Yes. No.

If Yes: Did having the option set email reminders for yourself help you incorporate the techniques into your routine? Yes. No.

If No: Did you use another type of reminder?

OVERALL EVALUATION

24. How user-friendly on a 0-10 scale (0, being the worst and 10 being the best possible score) would you rate the SPIN-SELF program?

25. Would you recommend this program to someone with scleroderma?

Yes. No.

If no, why?

26. What grade (on a 0-10 scale, 0 being the worst and 10 being the best possible score) would you give the program?

0 (worst) to 10 (best).

27. Is there anything you want to give us feedback about that was not included in this interview?
