# Supplementary material for: The Scleroderma Patient-Centered Intervention Network Self-Management Program: Protocol for a Randomized Feasibility Trial
Source: JMIR Res Protoc. 2020 Apr 24;9(4):e16799. doi: 10.2196/16799 (PMC7210498; doi:10.2196/16799)
Supplement: Multimedia Appendix 2 [file resprot_v9i4e16799_app2.pdf]

Canadian Institutes of Health Research | Instituts de recherche en santé du Canada

PROTECTED WHEN COMPLETED

Appl. #	363700

Notice of Decision - Thombs, Brett

Program: Project Grant
Competition: 201603PJT
Application Number: 363700
Title: Randomized Controlled Trial of an Internet-based Scleroderma Self-Management Program: A Scleroderma Patient-Centered Intervention Network (SPIN) Study

Applicants: Thombs, Brett

Boutron, Isabelle; Mouthon, Luc; Poiraudeau, Serge; Baron, Murray; Bartlett, Susan; et al

Research Institution: Jewish General Hospital (Montreal)/Hôpital général juif (Montréal)

Institution paid: Jewish General Hospital (Montreal)/Hôpital général juif (Montréal)

Competition Outcome:

Decision on your application: Approved
Approved Average Funding Amount: $561,909 **Term:** 3 **year(s)** 0 **month(s)**

Number of Applications Received at Stage 1: 3819
Number of Applications Accepted for Final Assessment Stage: 583
Number of Applications Approved: 491

Full competition results: http://www.cihr-irsc.gc.ca/e/49051.html

**Stage 1**
Decision on your application: Accepted for Stage 2
Consolidated Rank: 81.4280
Standard Deviation: 14.6488

Distribution of reviewer rankings

Percent	Number of Reviewers
90%-100%	1
80%-89%	2
70%-79%	1
60%-69%	1
50%-59%	0
0%-49%	0

CIHR IRSC

Institute of Aboriginal Peoples' Health
Institute of Aging
Institute of Cancer Research
Institute of Circulatory and Respiratory Health
Institute of Gender and Health
Institute of Genetics
Institute of Health Services and Policy Research
Institute of Human Development and Child and Youth Health
Institute of Infection and Immunity
Institute of Musculoskeletal Health and Arthritis
Institute of Neurosciences, Mental Health and Addiction
Institute of Nutrition, Metabolism and Diabetes
Institute of Population and Public Health

Institut de la santé des Autochtones
Institut du vieillissement
Institut du cancer
Institut de la santé circulatoire et respiratoire
Institut de la santé des femmes et des hommes
Institut de génétique
Institut des services et des politiques de la santé
Institut du développement et de la santé des enfants et des adolescents
Institut des maladies infectieuses et immunitaires
Institut de l'appareil locomoteur et de l'arthrite
Institut des neurosciences, de la santé mentale et des toxicomanies
Institut de la nutrition, du métabolisme et du diabète
Institut de la santé publique et des populations

July 15, 2016

Dr. Brett David Thombs
Institute of Community and Family Psychiatry
Jewish General Hospital
4333 Côte-Ste-Catherine Road
Montréal, Québec H3T 1E4

Dear Dr. Thombs,

On behalf of the Canadian Institutes of Health Research (CIHR), I am pleased to inform you that your recent application to the Project Grant – Spring 2016 competition entitled “Randomized Controlled Trial of an Internet-based Scleroderma Self-Management Program: A Scleroderma Patient-Centered Intervention Network (SPIN) Study” has been approved for funding.

Your application reviews and competition results can be accessed through ResearchNet. If you are unable to view these documents, please contact us at support@cihr-irsc.gc.ca. Your Authorization for Funding will follow in the mail.

As CIHR does not notify co-applicants of the decision, we ask that you inform those individuals involved, along with their research institutions (if different from your own) of the outcome of this application.

Should you have any questions, please do not hesitate to communicate with a Processing Officer in the Contact Centre at 613-954-1968 or by e-mail: support@cihr-irsc.gc.ca.

Congratulations on your success in this competition.

Sincerely,

Martine Lafrance

Martine Lafrance, Ph.D.
Manager, Project Grant Program/Investigator Initiated Research Branch
Research, Knowledge Translation and Ethics Portfolio

432976-201603PJT-PJT-363700-153261-DLPJT

Canadian Institutes of Health Research
Room 97, 160 Elgin Street, Address locator: 4809A
Ottawa, (Ontario) K1A 0W9 Tel.: (613) 941-2672
Fax (613) 954-1800 **www.cihr-irsc.gc.ca**

Instituts de recherche en santé du Canada
Pièce 97, 160 rue Elgin, Indice de l'adresse: 4809A
Ottawa, (Ontario) K1A 0W9 Tél.: (613) 941-2672
Fax (613) 954-1800 **www.irsc-cihr.gc.ca**

Canada

CIHR IRSC

Institute of Aboriginal Peoples' Health
Institute of Aging
Institute of Cancer Research
Institute of Circulatory and Respiratory Health
Institute of Gender and Health
Institute of Genetics
Institute of Health Services and Policy Research
Institute of Human Development and Child and Youth Health
Institute of Infection and Immunity
Institute of Musculoskeletal Health and Arthritis
Institute of Neurosciences, Mental Health and Addiction
Institute of Nutrition, Metabolism and Diabetes
Institute of Population and Public Health

Institut de la santé des Autochtones
Institut du vieillissement
Institut du cancer
Institut de la santé circulatoire et respiratoire
Institut de la santé des femmes et des hommes
Institut de génétique
Institut des services et des politiques de la santé
Institut du développement et de la santé des enfants et des adolescents
Institut des maladies infectieuses et immunitaires
Institut de l'appareil locomoteur et de l'arthrite
Institut des neurosciences, de la santé mentale et des toxicomanies
Institut de la nutrition, du métabolisme et du diabète
Institut de la santé publique et des populations

July 15, 2016

Dr. Brett David Thombs
Institute of Community and Family Psychiatry
Jewish General Hospital
4333 Côte-Ste-Catherine Road
Montréal, Québec H3T 1E4

Dear Dr. Thombs,

Congratulations on your success in the recent Canadian Institutes of Health Research (CIHR) Project Grant—Spring 2016 competition.

Your application was reviewed by your peers and considered to be of exceptionally high quality. You should take great pride in this achievement, particularly given the highly competitive nature of CIHR funding.

As you know, peer review is the cornerstone of our research funding system. This process is made possible because of the volunteerism of individuals who generously gave their time to review your application. We are continuously recruiting and retaining the most accomplished innovative and creative scientists to review health research proposals. As a CIHR-funded researcher, you may be invited to serve in the peer review process for future competitions.

To highlight your achievements and to communicate the value of health research to Canadians, we encourage you to work with your institution to promote your research. To support you in this activity, CIHR has developed guidelines on public communication available at: www.cihr-irsc.gc.ca/e/30789.html.

Once again, I offer you my congratulations and best wishes for success in your research.

Yours sincerely,

Alain Beaudet, MD, Ph.D.
President

433108-201603PJT-PJT-363700-153261-CLPJT

President

Canadian Institutes of Health Research
Room 97, 160 Elgin Street, Address locator: 4809A
Ottawa, (Ontario) K1A 0W9 Tel.: (613) 941-2672
Fax (613) 954-1800 **www.cihr-irsc.gc.ca**

Président

Instituts de recherche en santé du Canada
Pièce 97, 160 rue Elgin, Indice de l'adresse: 4809A
Ottawa, (Ontario) K1A 0W9 Tél.: (613) 941-2672
Fax (613) 954-1800 **www.irsc-cihr.gc.ca**

Canada

**Review Type / Type d'évaluation:**	Reviewer 1 / Évaluateur 1
**Name of Applicant / Nom du chercheur:**	Thombs,Brett
**Application No. / Numéro de demande:**	363700
**Agency / Agence:**	CIHR/IRSC
**Competition / Concours:**	Project Grant/Subvention Projet
**Committee / Comité:**	Project Grant Competition/Concours de subventions Projet
**Title / Titre:**	Randomized Controlled Trial of an Internet-based Scleroderma Self-Management Program: A Scleroderma Patient-Centered Intervention Network (SPIN) Study

Concept/Concept

Criterion/Critère: Quality of the Idea/Qualité de l'idée

Rating/Cote: G

Strengths/Forces: Self-efficacy in chronic disease (especially one as limited in direct therapeutic options) is clearly an area where care gaps exist.

Well-thought out approach, borrowing from experience in other chronic disease self-management initiatives/research/outcome.

Measurement plan considered.

Patient engagement in the development of the educational tool

Weaknesses/Faiblesses: rely on QoL measures - are they validated for the multiple issues the self-management modules address? Are disease- specific QoL measures better?

Self-efficacy is important - but other than QOL what other impacts might there be from improved self-efficacy? Does the cohort data permit measurement of health services use, functional abilities etc - which one would hope would be expected consequences of self-efficacy.

Criterion/Critère: Importance of the Idea/Importance de l'idée

Rating/Cote: E

Strengths/Forces: Patient ranked priority - and engagement in development emphasizes importance

Weaknesses/Faiblesses: Small number of patients might benefit from the intervention. Unlikely that other common chronic diseases will learn much? Is there potential that other rare diseases may benefit from this approach?

Feasibility/Faisabilité

Criterion/Critère: Approach/Approche

Rating/Cote: E+

Strengths/Forces: Straightforward approach and design.
PROM based.
Feasibility is good.
Connections would appear to support KT proposals

Weaknesses/Faiblesses: Not much information about the spectrum of Scleroderma as a disease and whether different complications or disease phenotype need to be considered in randomization or adaptation made to the outcome measure tools.

Criterion/Critère: Expertise, Experience and Resources/Expertise, expérience et ressources

Rating/Cote: O+

**Review Type / Type d'évaluation:**	Reviewer 1 / Évaluateur 1
**Name of Applicant / Nom du chercheur:**	Thombs,Brett
**Application No. / Numéro de demande:**	363700
**Agency / Agence:**	CIHR/IRSC
**Competition / Concours:**	Project Grant/Subvention Projet
**Committee / Comité:**	Project Grant Competition/Concours de subventions Projet
**Title / Titre:**	Randomized Controlled Trial of an Internet-based Scleroderma Self-Management Program: A Scleroderma Patient-Centered Intervention Network (SPIN) Study

Strengths/Forces: International team with a number of complementary skills. Team grant has allowed necessary infrastructure of the cohort to be created. Evidence of communication between members is obvious.
Seemingly good access to committed patient community and to the organizational collaboratives dealing with rare diseases and their needs.

Weaknesses/Faiblesses: No major issues

**Review Type / Type d'évaluation:**	Reviewer 2 / Évaluateur 2
**Name of Applicant / Nom du chercheur:**	Thombs,Brett
**Application No. / Numéro de demande:**	363700
**Agency / Agence:**	CIHR/IRSC
**Competition / Concours:**	Project Grant/Subvention Projet
**Committee / Comité:**	Project Grant Competition/Concours de subventions Projet
**Title / Titre:**	Randomized Controlled Trial of an Internet-based Scleroderma Self-Management Program: A Scleroderma Patient-Centered Intervention Network (SPIN) Study

Concept/Concept

Criterion/Critère: Quality of the Idea/Qualité de l'idée

Rating/Cote: E+

Strengths/Forces: This well-written proposal targets self-management efficacy enhancement for individuals living with systemic sclerosis (SSc). The team makes the argument that many approaches to self-management are generic in focus and rare disease populations have unmet needs, such as those living with SSc. A major strength is the excellent infrastructure and novel RCTs methods, embedded within the SPIN cohort.

Weaknesses/Faiblesses: While the infrastructure, methods, and a population addressed are innovative and novel, I find the trial outcomes somewhat underwhelming- I will elaborate further in the next sections.

Criterion/Critère: Importance of the Idea/Importance de l'idée

Rating/Cote: E++

Strengths/Forces: This section clearly outlines the burden of SSc in an informative and succinct manner. The pitfalls in the evaluation of current SSc approaches have been described, as well as the difficulty in recruiting people living with rare diseases into trials of SM programs. This logically leads into description of the fact the SPIN was formed by virtue of a CIHR team grant, which is excellent. The team approach to the novel cmRCT design in this context is very strong and efficient- this is commendable.

Weaknesses/Faiblesses: For this section, the drawback for me is the fact that the details around the prior development of the SM program and the feedback received are vague, which concerns me, moving into the RCT.

Feasibility/Faisabilité

Criterion/Critère: Approach/Approche

Rating/Cote: G

Strengths/Forces: The major strength to this proposal, as previously discussed, is the excellent and efficient infrastructure that has been created by virtue of SPIN. The RCT maneuvers are described clearly, as are the participants, the enrollment processes, and ethical considerations. The intervention components are thoughtful, as are the strategies to mitigate disappointment and related biases. Based on the proposed primary outcome, the sample size calculation is clear, as is feasibility (well explained). The approach to analyses are appropriate and the discussion of how the network is designed to overcome recruitment challenges is clear. The dissemination capacity is strong.

Weaknesses/Faiblesses: There are some issues which need to be addressed, in my view, to make this proposal stronger. First, as mentioned previously, the discussion on the prior development of the intervention and how feedback was received (and then used) is vague in the prior section and not mentioned in this one. To be comfortable in supporting this trial, I'd like to see clear, thorough plans for usability testing, feedback and iterations before moving into an effectiveness trial. This can be done all in the same proposal with a phased approach. If usability data have already been collected, it would be helpful to be referred to it to understand how user experience has been optimized and how problems have been addressed. I also find SM efficacy not the best choice of primary outcome. For the level of investment requested, I'd like to see this trial powered to detect differences in outcomes, such as healthcare utilization, that will have direct impact on health policy. Self-

efficacy is process related and indeed critical, but I find its lacks potential for real impact as a primary outcome. I was also surprised that I did not see a validated pain measure among the secondary outcomes, given how painful SSc has been argued to be (the case for that is strong). The fact that engagement with the components of SM will be measured is important but in reading the proposal I wondered about assessment of baseline digital literacy in relation to this. In addition, a cost effectiveness or utility analysis would also lend strength to the outcome definitions. Given how novel the SPIN network is, I also found the KT (not dissemination- that is strong) section of the proposal to be lacking in innovation. Intervention adherence strategies are not apparent.

Criterion/Critère: Expertise, Experience and Resources/Expertise, expérience et ressources

Rating/Cote: O++

Strengths/Forces: The team and related infrastructure are outstanding in my view.

Weaknesses/Faiblesses: No concerns.

**Review Type / Type d'évaluation:**	Reviewer 3 / Évaluateur 3
**Name of Applicant / Nom du chercheur:**	Thombs,Brett
**Application No. / Numéro de demande:**	363700
**Agency / Agence:**	CIHR/IRSC
**Competition / Concours:**	Project Grant/Subvention Projet
**Committee / Comité:**	Project Grant Competition/Concours de subventions Projet
**Title / Titre:**	Randomized Controlled Trial of an Internet-based Scleroderma Self-Management Program: A Scleroderma Patient-Centered Intervention Network (SPIN) Study

Concept/Concept

Criterion/Critère: Quality of the Idea/Qualité de l'idée

Rating/Cote: G

Strengths/Forces: The proposal is based on a sound and logical rationale – to provide pts with a rare disease with access to proven online self management resources

The overall goals and objectives of the project are well defined.

The unique aspect of the proposed project is the international team that has consolidated efforts to address the needs of a rare disease.

Weaknesses/Faiblesses: On line self management interventions are not novel and most on line self management models follow a similar format.

It is unknown how the modules were developed. ie. Is the content contained in the modules generic enough to be relevant and meaningful across countries, yet specific enough to address the needs of patients with scleroderma.

Criterion/Critère: Importance of the Idea/Importance de l'idée

Rating/Cote: E

Strengths/Forces: This study will likely offer a patient population access to important self care strategies and information that was previously inaccessible.

The SPIN cohort offers a unique method to test interventions on a larger scale to develop the evidence -base .

Weaknesses/Faiblesses:

Where did the need to design an online intervention emerge from? ie In previous work within SPIN , did patients identify that an online intervention was their preferred modality of receiving support ?

The online intervention appears to be targeted to patients with online skills and an interest to use the online self management program. Are there other interventions being developed to reach patients who are not interested in online interventions ?

How will the online intervention be sustained after the study if found to be effective?

Feasibility/Faisabilité

Criterion/Critère: Approach/Approche

Rating/Cote: E+

Strengths/Forces: The use of the cohort RCT design will enhances feasibility of conducting clinical trials within patient care, reduces loss of data, and address disappointment bias.

The proposed plan appears plausible in terms of patient recruitment ,i.e.. they need to recruit 1048 patients to complete the trial from a pool of 2000 currently enrolled pts in the SPIN cohort.

Weaknesses/Faiblesses: There are some methodological questions that were raised.

What is the percentage of patients who are NOT a part of the SPIN cohort?

Why target only patients with low self efficacy?

How useful will the intervention be to patients who are not web-savvy? Study excludes patients who do not go on the internet.

How will patients be motivated to complete the modules?

Are patients in the control group being asked about the types of support they are accessing ?

What measures are in place to address other biases such as co-intervention and contamination?

How to ensure that the effects of the intervention are attributed to the online modules and not the social support / emails that encourage exchange between patients?

Criterion/Critère: Expertise, Experience and Resources/Expertise, expérience et ressources

Rating/Cote: O

Strengths/Forces: A world wide collaboration of experts in scleroderma is involved in this study including 2000 pts by 2016, 100 clinicians from over 30 major centres.

There are opportunities included for doctoral studentships

Weaknesses/Faiblesses: Will the intervention be translated into other languages other than French?

If proven to be effective, how can the intervention be disseminated to patients without internet access?

**Review Type / Type d'évaluation:**	Reviewer 4 / Évaluateur 4
**Name of Applicant / Nom du chercheur:**	Thombs,Brett
**Application No. / Numéro de demande:**	363700
**Agency / Agence:**	CIHR/IRSC
**Competition / Concours:**	Project Grant/Subvention Projet
**Committee / Comité:**	Project Grant Competition/Concours de subventions Projet
**Title / Titre:**	Randomized Controlled Trial of an Internet-based Scleroderma Self-Management Program: A Scleroderma Patient-Centered Intervention Network (SPIN) Study

Concept/Concept

Criterion/Critère: Quality of the Idea/Qualité de l'idée

Rating/Cote: O+

Strengths/Forces: The project will study the effectiveness of a self-management intervention in patients with a rare chronic disease, systemic sclerosis. Novel aspects of the project include the intervention, which was designed in collaboration with investigators, patients, and other stakeholders, the use of the internet to complete the enrolment and randomize patients, deliver the intervention, and collect the data, and the use of a large international network to study an intervention in a rare disease.

The rationale is sound, the intervention has already been developed, and this project stems out of a successful previously funded one.

The proposal is well-written and clear.

Weaknesses/Faiblesses: The results of this study may not be able to be replicated in or transferable to other patient populations.

Criterion/Critère: Importance of the Idea/Importance de l'idée

Rating/Cote: E++

Strengths/Forces: Disease self-management is important and can have an effect on health care, health systems utilization, and health outcomes.
If successful, the results of this project could possibly inform development of instruments and evaluation of self-management in other chronic and/or rare diseases.
The impact of the studied disease on quality of life and health care utilization is important.

Weaknesses/Faiblesses: This is a rare disease, so the results of this project may not be generalizable to the population at large.

Feasibility/Faisabilité

Criterion/Critère: Approach/Approche

Rating/Cote: E++

Strengths/Forces: The approach is reasonable and some novel factors include the delivery of the intervention and collection of data via the internet, which allows for enrolment of patients on an international basis and for enrolment of the number of patients needed to complete the study given the rareness of systemic sclerosis.

Outcomes involve the use of validated questionnaires.

The intervention is already developed, the patient cohort is already assembled, and stakeholders have been involved in these steps.

The timeline is reasonable. Power calculations are reasonable and take into account attrition.

Weaknesses/Faiblesses: Outcomes directly measuring health outcomes such as medication use/adherence, disease progression/exacerbation, health care service utilization/hospitalization are not included in the scope of this project.

Criterion/Critère: Expertise, Experience and Resources/Expertise, expérience et ressources

Rating/Cote: O

Strengths/Forces: The principal researcher has a reasonable track record of publications and grant funding in this and other related research areas.

A multidisciplinary team has been established and statistical and other supports are in place.

Stakeholders have been involved in the development of the cohort and of the intervention.

A multi-national, multi-centre collaboration has been established.

Weaknesses/Faiblesses: No major weaknesses.

**Review Type / Type d'évaluation:**	Reviewer 5 / Évaluateur 5
**Name of Applicant / Nom du chercheur:**	Thombs,Brett
**Application No. / Numéro de demande:**	363700
**Agency / Agence:**	CIHR/IRSC
**Competition / Concours:**	Project Grant/Subvention Projet
**Committee / Comité:**	Project Grant Competition/Concours de subventions Projet
**Title / Titre:**	Randomized Controlled Trial of an Internet-based Scleroderma Self-Management Program: A Scleroderma Patient-Centered Intervention Network (SPIN) Study

Concept/Concept

Criterion/Critère: Quality of the Idea/Qualité de l'idée

Rating/Cote: O

Strengths/Forces: This is a strong proposal with a well written application, rationale and overall justification. The application proposes a pragmatic RCT to test the effect of adding a self-management program to usual care on disease management self-efficacy and health-related quality of life in patients with scleroderma.
The proposed RCT is anchored in The Scleroderma Patient-centered Intervention Network (SPIN, international) which was created to deal with challenges due to lack of N/power in such a rare condition. Importantly, the proposal follows a CIHR Team Grant which was awarded to create an infrastructure for trials of online disease-management tools for SSc (N=2000 patients in 2016, 440 will participate to the trial). SPIN uses a novel cohort-based trial design, in which patient outcome data are collected at regular intervals and trials are embedded in the cohort.
Applicants mention that the development, study of feasibility and testing of the proposed intervention has already been done. The intervention consists in an internet-based SM program on topics that were identified as relevant by the patients themselves in previous studies. The modules address 9 different targets.

Weaknesses/Faiblesses: My main question here is with the primary outcome chosen. The objective of a self-management program is ultimately to change the experience of symptoms and behaviours. Although global self-efficacy to self-manage is a relevant preliminary target, I wonder why specific clinical targets as related to modules were not included as outcomes. For example, fatigue, emotional distress, or sleeping difficulties are domains where validated measures exist. Wouldn't it be more clinically relevant to include these measures at least as secondary outcomes? (you could pick those who are more frequently reported by patients). Doing so, you could show that you empower patients and that this translates into relevant clinical outcomes. The current secondary outcome (HRQL) is a very distal target which is likely to be influenced by many other aspects, including relevant clinical outcomes (such as fatigue) identified by patients.

Criterion/Critère: Importance of the Idea/Importance de l'idée

Rating/Cote: O

Strengths/Forces: There is an important need to improve self-management in this population and to improve the way we test interventions in rare diseases. To date, only two pre-post intervention studies of SSc self-management programs have been conducted. This proposed trial will evaluate whether an online intervention can improve disease-management self-efficacy for patients with a rare disease without disease-modifying treatments. If effective, patient organization partners will disseminate the intervention to patients and healthcare providers. This could also serve as a prototype for similar programs in other rare diseases.

Weaknesses/Faiblesses: How self-management self-efficacy translates into clinical outcomes remains unknown. The clinical impact of the research could be improved if you replaced HRQL by more proximal outcomes (as secondary outcomes).

Feasibility/Faisabilité

Criterion/Critère: Approach/Approche

**Review Type / Type d'évaluation:**	Reviewer 5 / Évaluateur 5
**Name of Applicant / Nom du chercheur:**	Thombs,Brett
**Application No. / Numéro de demande:**	363700
**Agency / Agence:**	CIHR/IRSC
**Competition / Concours:**	Project Grant/Subvention Projet
**Committee / Comité:**	Project Grant Competition/Concours de subventions Projet
**Title / Titre:**	Randomized Controlled Trial of an Internet-based Scleroderma Self-Management Program: A Scleroderma Patient-Centered Intervention Network (SPIN) Study

Rating/Cote: E+

Strengths/Forces: This is a very powerful design as the trial is embedded in the cohort. Apparently patients who consented to be part of the cohort are more or less automatically proposed the trials (high feasibility)

The methods are very well written, and appropriate. This appears as a methodologically sound project. The intervention and the comparator are well defined, although the comparator could differ across countries (but this will be controlled for in analyses, the ability to control for that depends on N/site though).

Weaknesses/Faiblesses: I was puzzled by the fact that sample calculations are based on external research (meta-analysis of lay-led SM) and not the pre-test of the intervention. How can you guarantee you will have a significant clinical signal, even if you limit yourself to self-efficacy? The reading of the background gave me the feeling that standard proofs of concept (Phase II behavioural trials in the ORBIT model) were not performed. Similarly, it is unclear if the components of the intervention, and its dosage have been studied for their effects in past research. As it is, you do not give much argument to justify for a clinical signal of your intervention, as a simple preliminary pre-post should provide. I suggest this should be led prior to launching the full-scale trial.

The measure of self-efficacy to self-manage appears a very subjective evaluation of one’s capacity. With such a measure, positivity biases and expectations effects may be maximized in an unblinded trial, especially if the measure remains global and aggregates 6 different domains. Another limitation with this measure concern its relationship with the modules of the intervention. Logically one would expect direct links between the 6 items of the scale and the 9 domains of the intervention.

Although this issue was probably anticipated by the team, it is unclear how the applicants will deal with potential overlap of this RCT with other future RCT. You mentioned that the cohort participants will be routinely proposed enrollment in research. Here you plan to recruit roughly 25% of your cohort, so overlap is certain in the future. This could lead to important biases.

Criterion/Critère: Expertise, Experience and Resources/Expertise, expérience et ressources

Rating/Cote: O++

Strengths/Forces: This is a very strong team with previous collaboration. The team includes an impressive number of researchers and knowledge users. The project will be mostly managed by the PI and a post-doc fellow with appropriate expertise. SPIN benefits from >50 active members of the SPIN research collaboration, SPIN recruiting site personnel, and an outstanding, long-standing relationship with key patient organizations. SPIN activities are jointly overseen by its Steering Committee and Patient Advisory Committee. SPIN Project Teams, including the SPIN-SM Team, are dedicated to the development, testing and dissemination of SPIN’s online tools. They are supported by SPIN Core Teams, including Core Teams in Information Technology, Data Management, Research Methods and Biostatistics, Measurement, Health Economics, Bioethics, and Knowledge Transfer. This appears as a well-organized research team.

The PI is a psychologist, mid-career researcher and founder and director of SPIN. He researches disease-management strategies to improve quality of life and reduce disability among people with rare diseases, including sclerodermia. He has been the PI on 5 CIHR-funded meetings that brought together the international scleroderma community and resulted in the creation of SPIN. SPIN was funded by CIHR (PI – Thombs; $1,499,765; 2012-2017) to use innovative methods to conduct high-quality clinical trials embedded in an ongoing cohort. The PI has extensive experience in several areas relevant to this application including research methods and evidence synthesis. He has authored 60 publications on sclerodermia.

Excellent environment.

Weaknesses/Faiblesses: None

CIHR IRSC

NOTE

Your application was assessed by the Stage 1 reviewers. Based on the ranking of your application relative to the other applications in the competition, it was identified as highly competitive and did not require further discussion by the Final Assessment Stage committee. As a result, no Scientific Officer notes were generated. The reviewer reports from Stage 1 are available on ResearchNet.

Please refer to the *Notice of Decision* for more information regarding the ranking of your application.

For information regarding the Project Grant Program peer review process, please refer to the *Peer Review Manual - Project* (http://www.cihr-irsc.gc.ca/e/49564.html).

Votre demande a été évaluée par les évaluateurs de l’Étape 1. Compte tenu du classement de votre demande par rapport aux autres demandes dans le concours, celle-ci a été identifiée comme étant très compétitive et ne nécessitant pas de discussion par le comité de l’étape d’évaluation finale. Par conséquent, il n’y a pas de notes de l’agent scientifique. Les rapports d’évaluation de l’Étape 1 sont disponibles sur RechercheNet.

Veuillez consulter l’avis de décision pour plus d’information sur le rang de votre demande.

Pour plus d’information sur le processus d’évaluation par les pairs du Programme de subventions Projet, veuillez consulter le *Guide d’évaluation par les pairs – Projet* (http://www.cihr-irsc.gc.ca/f/49564.html).
